# *slendr*: a framework for spatio-temporal population genomic simulations on geographic landscapes

**DOI:** 10.24072/pcjournal.354

**Published:** 2023-12-15

**Authors:** Martin Petr, Benjamin C. Haller, Peter L. Ralph, Fernando Racimo

**Affiliations:** 1 Lundbeck Foundation GeoGenetics Centre, Globe Institute, University of Copenhagen, Denmark; 2 Section for Molecular Ecology and Evolution, Globe Institute, University of Copenhagen, Denmark; 3 Department of Computational Biology, Cornell University, Ithaca, NY, USA; 4 Institute of Ecology and Evolution, University of Oregon, Eugene, OR, USA

## Abstract

One of the goals of population genetics is to understand how evolutionary forces shape patterns of genetic variation over time. However, because populations evolve across both time and space, most evolutionary processes also have an important spatial component, acting through phenomena such as isolation by distance, local mate choice, or uneven distribution of resources. This spatial dimension is often neglected, partly due to the lack of tools specifically designed for building and evaluating complex spatio-temporal population genetic models. To address this methodological gap, we present a new framework for simulating spatially-explicit genomic data, implemented in a new R package called *slendr* (www.slendr.net), which leverages a SLiM simulation back-end script bundled with the package. With this framework, the users can programmatically and visually encode spatial population ranges and their temporal dynamics (i.e., population displacements, expansions, and contractions) either on real Earth landscapes or on abstract custom maps, and schedule splits and gene-flow events between populations using a straightforward declarative language. Additionally, *slendr* can simulate data from traditional, non-spatial models, either with SLiM or using an alternative built-in coalescent *msprime* back end. Together with its R-idiomatic interface to the *tskit* library for tree-sequence processing and analysis, *slendr* opens up the possibility of performing efficient, reproducible simulations of spatio-temporal genomic data entirely within the R environment, leveraging its wealth of libraries for geospatial data analysis, statistics, and visualization. Here, we present the design of the *slendr* R package and demonstrate its features on several practical example workflows.

## Introduction

Most evolutionary processes in nature have a spatial dimension. Indeed, since its beginnings, the field of population genetics has aspired to build interpretable models of spatial population dynamics (Guillot et al., 2009; Barton et al., 2013). These include classic theoretical models such as Fisher’s wave-of-advance model (Fisher, 1937), Wright’s isolation-by-distance model (Wright, 1943), Kimura’s stepping-stone model (Safner et al., 2011; Petkova et al., 2016; Ringbauer et al., 2018; Al-Asadi et al., 2019; Marcus et al., 2021), and Malecot’s lattice model (Malécot, 1951; Nagylaki, 1976; Rousset, 1997). The field also has a long history of modeling continuous spatial genetic variation (Levene, 1953; Slatkin, 1973; Barton, 1979; Beerli & Felsenstein, 2001; McRae, 2006; Duforet-Frebourg & Blum, 2014; Bradburd et al., 2018), inferring spatial covariates associated with genetic patterns (Hanks & Hooten, 2013) and detecting spatial barriers to migration (Safner et al., 2011; Petkova et al., 2016; Ringbauer et al., 2018; Al-Asadi et al., 2019; Marcus et al., 2021). However, these latter efforts are hampered by a lack of good theoretical predictions for continuous, two-dimensional models (Felsenstein, 1975; Barton et al., 2002), and simulations can provide a valuable tool in the absence of analytical theory.

The dramatic increase in the number of published whole-genome sequences in the last 20 years (The 1000 Genomes Project Consortium, 2010; Mallick et al., 2016; Palkopoulou et al., 2018; Feuerborn et al., 2021), and the advent of ancient genomics (Green et al., 2010; Rasmussen et al., 2010), have revealed previously unknown migration events in the history of several species, such as dogs (Bergström et al., 2020), horses (Librado et al., 2021), elephantids (Meyer et al., 2017), and humans (Lazaridis et al., 2014; Fu et al., 2016). Since migration of populations involves spatial displacement, populations trace their ancestry to different geographic locations (Ralph & Coop, 2013; Osmond & Coop, 2021; Wohns et al., 2022). In the context of human history, processes including past migration, gene flow, and population turnovers have been shown to have had a major influence on the present-day distribution of genomic variation (Pickrell & Reich, 2014; Slatkin & Racimo, 2016). Properly anchoring these past demographic events in both time and space has been a focus for new modeling approaches (Racimo et al., 2020; Osmond & Coop, 2021; Wohns et al., 2022), and is a question of high interest not only in genetics (Bradburd & Ralph, 2019) but also in ecology (Frachetti et al., 2017; Loog et al., 2017; Delser et al., 2021; Crabtree et al., 2021).

Despite the key role of geography in population genetics, tools specifically designed for describing and simulating complex spatio-temporal processes are still lacking. Spatial simulations are important not just for rigorous testing and evaluation of existing inference tools and facilitating the development of new inference methods (Liu et al., 2006; Currat & Excoffier, 2011; Delser et al., 2021; Osmond & Coop, 2021; Wohns et al., 2022), but also for gaining intuition about the expected behavior of the processes influencing the patterns of genetic variation under various scenarios of spatial population dynamics (Felsenstein, 1975; Slatkin & Excoffier, 2012). While powerful simulation approaches based on coalescent theory have been developed (Hudson, 2002; Ewing & Hermisson, 2010; Staab et al., 2015; Kelleher et al., 2016), these have little or no notion of spatiality due to fundamental obstacles to incorporating space into the coalescent framework (Barton et al., 2002, 2010, 2013), although recent algorithmic advances are promising (Kelleher et al., 2014). The first pioneering attempt at simulating spatial population genetic data was the software package SPLATCHE (Currat et al., 2004, 2019). However, SPLATCHE’s simulation engine is limited to discrete demes based on the stepping-stone model, allows simulation of no more than two populations co-existing at a time, and is not suitable for simulating sequence data at a whole-genomic scale (Currat et al., 2019). The most advanced simulator with spatial capabilities is currently the forward population genetic simulation framework SLiM (Haller & Messer, 2017, 2019). Highly popular in the population genetics community, SLiM contains a vast library of features for simulating individuals in continuous space (as opposed to older approaches based on discrete demes), including spatial interactions between individuals, neighborhood-based mate selection, and customisable offspring dispersal (Haller & Messer, 2019). Moreover, the recent implementation of tree-sequence recording in SLiM has opened up the possibility of efficient simulation of massive genome-scale and population-scale datasets (Haller et al., 2019).

Despite these advances in population genetic simulations, geospatial data analysis remains a complex field with a steep learning curve. Performing even basic manipulations of spatial cartographic objects, handling diverse data formats, and transforming data between different projections and coordinate reference systems (CRS) requires a non-trivial amount of domain-specific knowledge (Lovelace et al., 2019). Moreover, because the technicalities of geospatial computation are generally not within the scope of population genetic software, available tools do not provide dedicated functionality for building complex and dynamic spatial population models in a straightforward manner. Developing such models and simulating data from them currently requires hundreds of lines of custom code, which is error-prone and hinders reproducibility. Additionally, the lack of specific frameworks for analyzing and visualizing spatially-explicit genomic data further hinders the methodological and empirical progress in spatial population genetics. A flexible and easy-to-use simulation framework specifically designed for developing spatio-temporal population models and analyzing spatial genomic data would expand the horizons of the field, allowing researchers to evaluate the accuracy of novel spatial methods, to test detailed hypotheses about demography and selection, and to answer entirely new kinds of questions about the interactions between organisms across space and time. For instance, many conceptual models and visualizations of past migration events involve depictions of movements of large population ranges across a map as various environmental or cultural conditions change; however, there is currently no easy way to simulate these movements and generate realistic spatio-temporal genomic data.

To address these issues, we have developed a new programming framework, called *slendr*, designed for simulating and analyzing spatially-explicit genomic data (available at www.slendr.net with extensive documentation and tutorials). The core component of this framework is an R package which leverages real Earth cartographic data (or, alternatively, an abstract user-defined spatial landscape) to programmatically and visually encode spatial population boundaries and their temporal dynamics across time and space, including expansions, migrations, population splits, and gene flow. Because of the challenges involved in testing and validating complex models, *slendr* encourages an interactive workflow in which each component of the model can be inspected and visualized as the model is incrementally constructed in a “bottom-up” fashion. Spatio-temporal models programmed in *slendr* can then be executed using a SLiM back-end script which is bundled with the package and can be controlled by a dedicated R function without leaving the R environment. Additionally, traditional, random-mating, discrete-deme, non-spatial population models can also be simulated, either in forward time using the aforementioned SLiM script or using an alternative coalescent *msprime* (Baumdicker et al., 2022) back-end script which is also bundled with the R package and can provide a more efficient simulation engine for non-spatial models. Both simulation engines of *slendr* save genomic outputs in the form of an efficient tree-sequence data structure (Kelleher et al., 2018), and the *slendr* R package provides a set of functions for loading and processing tree-sequence output files and computing population statistics on them by seamlessly integrating the *tskit* tree-sequence analysis Python module into its R interface. Additional functionality includes conversion of individual trees to a standard R *ape* phylogenetic format (Paradis & Schliep, 2019), and automatic transformation of spatial tree-sequence table data to the standardized *sf* format for geospatial data analysis in R (Pebesma, 2018).

Overall, the *slendr* R package facilitates reproducibility by providing a unified framework for writing complete spatial simulation and analysis pipelines entirely in R, which we demonstrate with several concrete examples.

## Design of *slendr*

From a software design perspective, the *slendr* R package represents a tight integration of three distinct parts. First, it implements an interactive and visually-focused R interface for encoding spatio-temporal population dynamics focused on building arbitrarily complex models from small individual components (i.e., simple R objects), designed to require only a minimum amount of code. Second, *slendr* includes two back-end simulation scripts implemented in SLiM (Haller & Messer, 2019) and *msprime* (Baumdicker et al., 2022). These scripts are bundled with *slendr*, are specifically tailored to interpret slendr demographic models, and produce tree-sequence files as output (Haller et al., 2019). Lastly, *slendr* provides an interface to the *tskit* tree-sequence analysis library (Kelleher et al., 2018). Although this library is written in C and Python, *slendr* exposes its functionality to the R environment in an R-idiomatic way, blending it naturally with the popular “tidyverse” philosophy of data analysis (Wickham et al., 2019).

**Figure 1 -:**
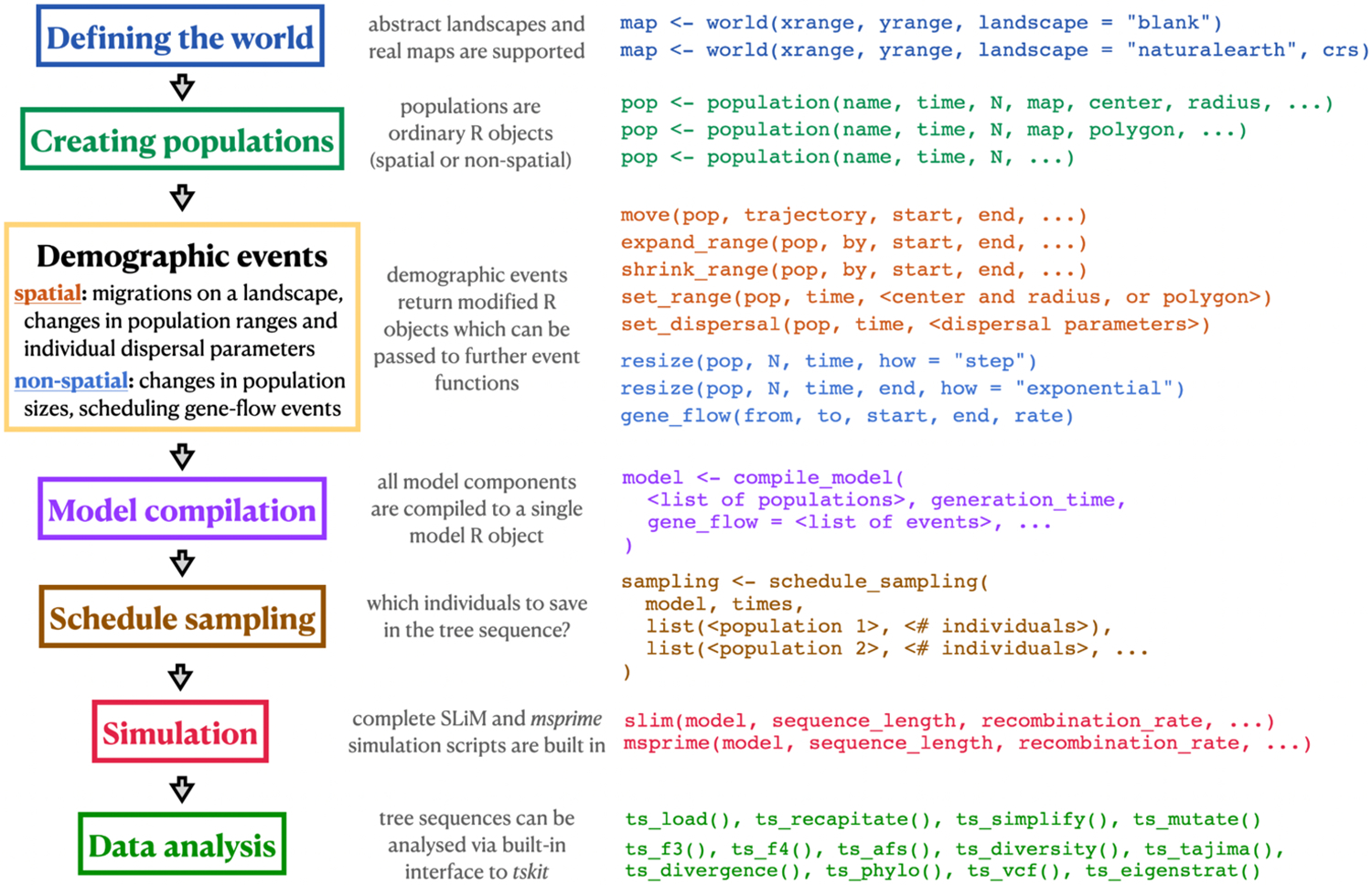
Schematic overview of a hypothetical *slendr* simulation and analysis workflow. The colored rectangles on the left indicate individual steps of a hypothetical *slendr* workflow. Short code snippets in matching colors on the right show examples of *slendr*’s declarative interface used in each step, focusing only on a selected few relevant functions and their most important arguments (additional optional arguments are replaced by the “…” ellipsis symbol). The full function reference index can be found at http://www.slendr.net/reference. Note that regardless of whether a spatial or non-spatial *slendr* model is being defined and simulated, the workflow remains identical: the same functions are used for both types of models, and the spatial or non-spatial nature of a model is automatically detected by *slendr*.

Although these three parts operate at fundamentally different levels under the hood, this integrated approach allows all steps of a *slendr* workflow—from specifying spatio-temporal demographic models, to executing simulations and analyzing simulation results—to be performed without leaving the R environment (Figure 1). This allows the user to leverage R’s features for visualization and interactive data analysis at every step of the analytic pipeline, and facilitates reproducibility by eliminating the need to manually integrate disparate software tools and programming languages (Sandve et al., 2013). In this way, *slendr* follows the footsteps of the original design of the S (and later R) languages: to present a consistent and convenient data-analysis–focused domain-specific front end to more efficient and faster tools written in other languages and frameworks (in this case SLiM and *msprime*) (Chambers, 2020).

In the remainder of this section we outline the individual steps of a typical *slendr* simulation and analysis workflow, as well as describe the individual building blocks of the three main components of the *slendr* framework mentioned above.

### Defining the world

At the beginning of a *slendr* workflow, the user defines the parameters of the world that the simulation will occupy using the function world() (Figure 1). If the simulated world represents a region on Earth, the appropriate set of vectorised spatial features will be automatically downloaded from a public-domain cartographic database (www.naturalearthdata.com). The user can also specify a dedicated coordinate reference system (CRS) appropriate for the projection of the geographic region of interest in order to minimize the distortion of distances and shapes inherent to transforming geometries (in this case population ranges and landscape features) from the three-dimensional Earth surface to its two-dimensional representation on a map. Alternatively, the world can be represented by an abstract landscape, optionally with custom features such as islands, barriers, or corridors. If a non-spatial deme-based model is to be simulated, this step can be omitted and no changes to the downstream steps described below are needed.

### Creating populations and scheduling demographic events

Populations in *slendr* are created with the population() function which creates a simple R object containing the parameters of the population that was created (Figure 1). In addition to specifying the name, time of appearance, and initial number of individuals for the new population, the user can also specify a world object and, if desired, a set of coordinates for the spatial range that the population will occupy. For convenience, the coordinates of all spatial objects in *slendr* (maps, geographic regions, population ranges) are always specified in the global geographical CRS (i.e., degrees of longitude and latitude) but are then automatically internally transformed into the chosen projected CRS (which uses units of meters) if it was specified when creating the world (Figure 1). This way, users can encode spatial coordinates in familiar units of longitude and latitude while *slendr* internally maintains the proper shapes and distances of spatial features by performing all spatial transformations in the projected CRS.

All *slendr* spatial objects are internally represented using a data type implemented by the R package *sf* (Pebesma, 2018), which has emerged as the de facto standard for geospatial data analysis in R (Lovelace et al., 2019). Despite the convenience of the *sf* framework, manipulation of geospatial objects in *sf* still requires writing a non-trivial amount of code dealing with low-level technical details (manipulating and transforming the coordinates of points, lines and polygons). Because most of these technical details are not relevant for specifying population genetic models, we designed a set of domain-specific functions for encoding spatial population dynamics which are expressed in terms of population genetics concepts rather than geometric transformations (Figure 1). For instance, the move() function accepts a *slendr* population object (i.e., internally an *sf* object, encapsulating the low-level geometric coordinates of the population), a trajectory given as a list of coordinates in longitude and latitude, and a timespan over which the population displacement should occur (Example 3 and Figure 4). Other kinds of dynamic spatial events (population range expansions and contractions, for example) are implemented in an analogous manner. Other demographic events, such as population size changes and gene flow, can be scheduled similarly with another set of straightforward functions (Figure 1).

For spatial models, the user has the option to fine-tune the within-population individual dispersal and mating dynamics (described in detail in Example 2 and Figure 3) using a set of parameters such as the maximum mating distance between individuals, the dispersal distance of offspring from their parents (and the kernel function of this dispersal), or the parameter influencing the uniformity of the dispersal of individuals within their population’s spatial boundary. These can be assigned for each population separately or kept at their default values given in the compile_model() step (as we show in Example 2). The competition parameter determines the maximum neighborhood distance in which individuals in a SLiM simulation compete with each other for space. If this distance is small, then individuals with nearby neighbors have much lower fitness. If the distance is larger, then the effects of crowding are more diffuse. However, if this distance is larger than the dispersal distance (as in Example 2), populations tend to self-organize into an evenly-spaced grid of patches (Figure 3C). Using the competition parameter, within-population dynamics can thus be fine-tuned to represent various levels of individual clustering into sub-groups (Figure 3C). In addition to the competition parameter, a mating parameter determines the maximum distance to which an individual will look for a mate to produce offspring. Finally, a dispersal parameter determines how far an offspring can end up from its parent, and a related dispersal_fun argument characterizes the density function for this dispersal: “normal” (default), “uniform”, “cauchy”, “exponential”, or “brownian”; more details are available in the *slendr* R package documentation at http://www.slendr.net/reference. We note that changes in all three spatial interaction and dispersal parameters can be also scheduled dynamically at specific times throughout the run of a model with a *slendr* function set_dispersal().

A standard feature of many population genetic frameworks is the specification of the times of various demographic events in terms of generations, either forwards in time starting from generation 1 (as is the case with SLiM) or backwards in time starting from time 0 “in the present” (as is the case with coalescent frameworks such as *msprime*). This can be cumbersome in cases when the events or samples of interest are traditionally specified in times of “years before present” (such as dated ancient DNA samples), or in situations in which it would be desirable to simulate future outcomes, as in ecological predictive modeling. Moreover, because these standard times often need to be converted into generations by a factor specifying the length of the generation time of the species of interest, this can easily lead to frustrating bugs in simulation scripts. To ameliorate this situation, *slendr* allows the users to specify times in whichever time units they would prefer, in either the forward or backward direction. The time direction is automatically detected by *slendr* from the sequence of demographic events specified for a model (but can also be set explicitly), and the conversion of event times into generations is performed in the compilation step via the provided generation_time argument to compile_model() (described below). Similarly, times of the tree-sequence nodes in *slendr*’s outputs (which are specified by most simulation software in terms of generations backwards in time) are automatically converted by *slendr* back into the units of time used by the user during model specification.

Because every *slendr* demographic event function returns a modified population object which can be further used as an input to other *slendr* functions, the R interface encourages a workflow in which complex models are composed incrementally from smaller components (Figure 1, Examples 1–3). Importantly, because each *slendr* function assures the consistency of the model by enforcing appropriate constraints during the model definition process (e.g., a population cannot be moved or participate in a gene-flow event at a time when it would not yet exist), this workflow facilitates the early discovery of bugs before the simulation (which can be extremely computationally costly) is even executed. This is further facilitated by a convenient set of plotting functions, such as plot_map() and plot_model(), which can visualize the spatio-temporal dynamics of the specified model (or its individual components) as the model is being incrementally developed.

### Model compilation

Having defined all the individual components of a population model (i.e., created all the necessary population and gene-flow events), the user calls the function compile_model() to compile the model configuration to a single R object (Figure 1)—a step in which *slendr* performs additional checks for model consistency and correctness. Furthermore, this operation also transforms the model components from their R representation into a set of files on disk, written in a format interpretable by the built-in SLiM and *msprime* simulation back-end scripts which are used to execute *slendr* models in the next phase, as described below. The compiled model object can also be used as input for a built-in R-based interactive browser app built using the *shiny* R package (Chang et al., 2023) which allows the user to “play” the defined spatial model dynamics over time and explore the “admixture graph” implied by the model (Patterson et al., 2012) for additional verification of the model’s correctness. The functions plot_map() and plot_model() mentioned above also accept a compiled model object as their input and produce a static visualization of the model.

### Scheduling sampling events and simulation

The *slendr* package comes bundled with two simulation back-end scripts which were tailored to interpret the configuration files produced by the compile_model() function and simulate the model, triggering all of the encoded population dynamics in the course of the simulation run.

The first back-end script is written in SLiM’s programming language Eidos (Haller & Messer, 2019), and can execute both spatial and non-spatial *slendr* models in a Wright–Fisher setting by calling *slendr*’s slim() function. The second back-end script is implemented using *msprime* (Baumdicker et al., 2022) and is designed to interpret the compiled *slendr* model in a non-spatial setting as a standard coalescent simulation by calling *slendr*’s msprime() function. Both simulation engines can interpret the same *slendr* model without a need to make any changes. For instance, a spatial model can be run with the *msprime* back end, in which case the spatial component of the model is simply ignored. Because coalescent simulations are generally much more computationally efficient than their forward-time counterparts, the *msprime* back end of *slendr* can be useful for R users who would like to run a large number of traditional, non-spatial simulation replicates efficiently without having to write custom Python *msprime* code or use its *ms*-like command-line interface (Hudson, 2002). Importantly, the correctness of both *slendr* simulation engines is validated using a set of automatic statistical tests on non-spatial models which ensure that when a *slendr* model is run in both SLiM and *msprime*, the demographic events specified by the model (population splits, population size changes, and gene-flow events) result in equivalent site-frequency spectra and *f*-statistics (Patterson et al., 2012) between both back ends.

Leveraging the ability to save simulation outputs as a tree sequence (Kelleher et al., 2019; Speidel et al., 2019) from both SLiM (Haller et al., 2019) and *msprime* (Baumdicker et al., 2022), *slendr* embraces the tree sequence as its primary output format. This is powerful not only because the tree sequence represents an extremely efficient representation of even large-scale population genomic data, but also because it provides an elegant way to calculate many population genetic statistics of interest, a feature which we describe in more detail in the next section. To specify which simulated individuals should be recorded in the output tree sequence, *slendr* provides two alternative approaches. First, if no explicit sampling schedule is specified, all individuals living at the very end of a SLiM simulation run are explicitly sampled (i.e., “remembered”) in the tree sequence output, matching the default behavior of SLiM. If a *slendr* model is simulated with the *msprime* back end, the number of recorded individuals will be equal to the population size of each population at the start of the coalescent process looking backwards in time (i.e., in “the present”). Alternatively, *slendr* provides a flexible way to trigger sampling events via its schedule_sampling() function, which allows one to specify the time (and, optionally, the location) at which a sample comprising a given number of individuals from a given population should be taken and recorded in the tree sequence (*Example 3*). To improve readability and interpretation of *slendr* analysis code, every sampled individual can be referred to using its readable name during tree-sequence processing and computation of statistics (*Examples 1, 2, and 4)* rather than just by numeric identifiers as is the case with the default tree-sequence analysis workflow with *tskit* (Kelleher et al., 2018).

### Data analysis

The default output of a *slendr* simulation is a tree sequence. However, because processing and analysis of tree-sequence files requires a non-trivial knowledge of Python or C (Kelleher et al., 2018) which many R users might not have, *slendr* provides an R-idiomatic interface to the most commonly used *tskit* tree-sequence methods such as the allele frequency spectrum, Patterson’s *f*-statistics, and various summary statistics of population diversity (Patterson et al., 2012; Ralph et al., 2020). This way, users can design population genetic models in R, execute them from R using the built-in slim() or msprime() functions, and analyze the resulting tree sequence data without having to leave the R environment for downstream statistical analyses and plotting, and without the need to convert outputs to other bioinformatic or population genetic file formats. Although primarily designed for analysis of tree sequences generated from *slendr* models, the R-*tskit* interface can operate also on tree sequences without *slendr*-specific metadata. Therefore, users who would prefer to run simulations with standard *msprime* or SLiM scripts but are interested in analyzing their tree-sequence results in R will still find the *slendr* R package useful. The reference manual at http://www.slendr.net/reference contains a complete list of *tskit* tree-sequence methods that have been integrated into *slendr*’s R interface. If integration with traditional tools such as PLINK (Purcell et al., 2007) or ADMIXTOOLS (Patterson et al., 2012) is required, functions for exporting to VCF (Danecek et al., 2011) and EIGENSTRAT (Patterson et al., 2012) are also provided.

During a spatial simulation in SLiM, each sampled individual’s location on the simulated landscape is tracked and recorded in the tree sequence, encapsulating the full spatio-temporal genealogical history that has been simulated. When the tree-sequence output file is then loaded by *slendr*, *slendr* processes the spatial locations of nodes in the tree sequence (which represent chromosomes of past and present individuals), and transforms them back into the original coordinate system of the simulated world, adding additional annotation data such as readable names of sampled individuals, population assignments of each individual and node, etc. Furthermore, this information is exposed in an *sf*-compatible format, meaning that the spatio-temporal information about ancestral relationships between simulated samples can be processed, analyzed, and visualized using a wide range of R packages including *sf*, *ggplot2*, and *dplyr* (Pebesma, 2018; Wickham et al., 2019). Additionally, individual trees in the tree sequence can be extracted by a *slendr* function, ts_phylo(), which converts *tskit*-formatted tree objects into the format defined by the R phylogenetics package *ape*, which has been the standard for phylogenetics in the R ecosystem for nearly two decades (Paradis & Schliep, 2019). This gives *slendr* users even more options to analyze tree-sequence results with a large array of standard phylogenetics tools available for the R environment (Paradis, 2011).

### Installation and software dependencies

*slendr* is currently developed for macOS and Linux. It is available on the CRAN R package repository at https://CRAN.R-project.org/package=slendr, and can be installed from the interactive R console with the standard command install.packages(“slendr”). Development versions of *slendr* which contain latest bug fixes and new experimental features can be installed from its GitHub repository using the R package *devtools* with the R command devtools::install_github(“bodkan/slendr”).

Two external software dependencies must be present on a user’s system to leverage the full functionality of *slendr*: a forward population genetic simulator SLiM (Haller & Messer, 2019) (which is required for running spatial simulations and non-spatial simulations in the forward-time setting) and a trio of Python modules *msprime* (Baumdicker et al., 2022), *tskit* (Kelleher et al., 2018) and *pyslim* (https://github.com/tskit-dev/pyslim) (which are needed to run *slendr* models as coalescent simulations and to analyze tree-sequence data).

The SLiM software is available for all major operating systems and its installation instructions can be found at https://messerlab.org/slim/. Importantly, the current version of *slendr* requires the latest release of SLiM 4.0. In order to use SLiM for simulations in *slendr*, the R session needs to be aware of the path to the directory containing the SLiM binary. Calling library(slendr) for the first time provides an informative message for the user on how this can be accomplished by modifying the $PATH variable by editing the ~/.Renviron file.

Because some users might find the experience of setting up a dedicated Python environment with the necessary Python modules challenging (especially users who exclusively work with R), *slendr* provides an R function setup_env() which automatically downloads a completely separate Python distribution and installs the required versions of *tskit*, *msprime*, and *pyslim* Python modules in their correct required versions into a dedicated virtual environment without any need for user intervention. Moreover, this Python installation and virtual environment are isolated from other Python configurations that might be already present on the user’s system, thus avoiding potential conflicts with the versions of Python and Python modules required by *slendr*. Once this isolated Python environment is created by setup_env(), users can activate it in future R sessions by calling a helper function init_env() after loading *slendr* via library(slendr). Therefore, although *slendr* uses Python modules for internal handling of tree-sequence data and coalescent simulation, direct interaction with Python is not necessary.

### Relationship of *slendr* to SLiM and *msprime*

Given that *slendr*’s simulation engines are implemented in SLiM and *msprime*, it is worth elaborating on its relationship to these simulation frameworks, particularly in terms of the features supported by *slendr*. First, it is important to note that *slendr* is not simply a wrapper for SLiM and *msprime* in the strict sense of the word, since *slendr* does not provide an R equivalent of every function and method provided by SLiM and *msprime*. Instead, *slendr* aims to provide a user-friendly, R-idiomatic way to encode a particular class of “traditional” Wright-Fisher population genetic models frequently used in evolutionary biology and population genetics, allowing users to employ such models with a minimal amount of coding. Most importantly, *slendr* models currently assume that populations evolve via random mating, and that the genomes of individuals evolve neutrally, with mutations overlaid on top of the simulated genealogies after each simulation run. This applies also to spatial *slendr* demographic models, with the caveat that interaction and dispersal distance parameters can—depending on the exact parametrization of each spatial *slendr* model—cause individuals to only mate locally, which can have interesting implications for the behavior of standard population genetic statistics (as shown in Example 2).

The complete set of models supported by *slendr* is likely to slightly expand over time as new features are implemented. Details of new features, such as customized recombination maps and non-neutral mutation types, are being discussed with the community on the GitHub page of *slendr* (https://github.com/bodkan/slendr), and users are encouraged to provide feedback there. The four practical examples (Examples 1–4 below) have been designed to demonstrate the full range of *slendr*’s features at the time of writing.

Finally, because *slendr*’s forward and coalescent simulation back ends are implemented as fairly standard SLiM and *msprime* scripts, the performance of *slendr* simulations and tree-sequence analyses can be assessed using already-existing benchmarks and guidelines provided by publications describing SLiM and *msprime* (Haller et al., 2019; Baumdicker et al., 2022; Haller & Messer, 2023).

## Practical examples

In the following sections, we present the features of the *slendr* R package with several practical examples, each of which focuses on a different aspect of the *slendr* simulation framework. We start by showing how traditional, non-spatial, random-mating models can be specified with a minimum amount of R code (*Example 1*). We then proceed with two examples of spatial models: first, a model showing how the degree of the spatial spread of a population can be adjusted by setting the within-population individual-based dispersal dynamics (*Example 2*); second, a model which schedules the movements of entire population ranges across a landscape (*Example 3*). These examples are intended to demonstrate *slendr*’s ability to define complex spatio-temporal models incrementally, building them from simpler components. We also emphasize how *slendr* model configuration and simulation steps naturally flow into data analysis, all within the R environment. In the final demonstration (*Example 4*), we tap into the rich information embedded in spatial tree sequences to visualize individual trees on a landscape, tracing the complex spatio-temporal ancestry of an individual on the simulated map. Extended versions of these and many other examples with complete reproducible code for simulation, analysis, and plotting can be found as standard R package vignettes at *slendr*’s website (www.slendr.net).

### Example 1: Traditional non-spatial model

Regardless of whether a spatial or non-spatial model is defined and simulated, the *slendr* workflow remains the same. Therefore, before we explore spatial models, we begin by showing how a traditional, non-spatial population genetic model can be constructed with *slendr* and how users can compute population genetic statistics on simulated tree-sequence outputs using *slendr*’s R interface to the *tskit* tree-sequence analysis library (Kelleher et al., 2018) (represented by functions with the ts_*() prefix, Figure 1).

**Figure 2 -:**
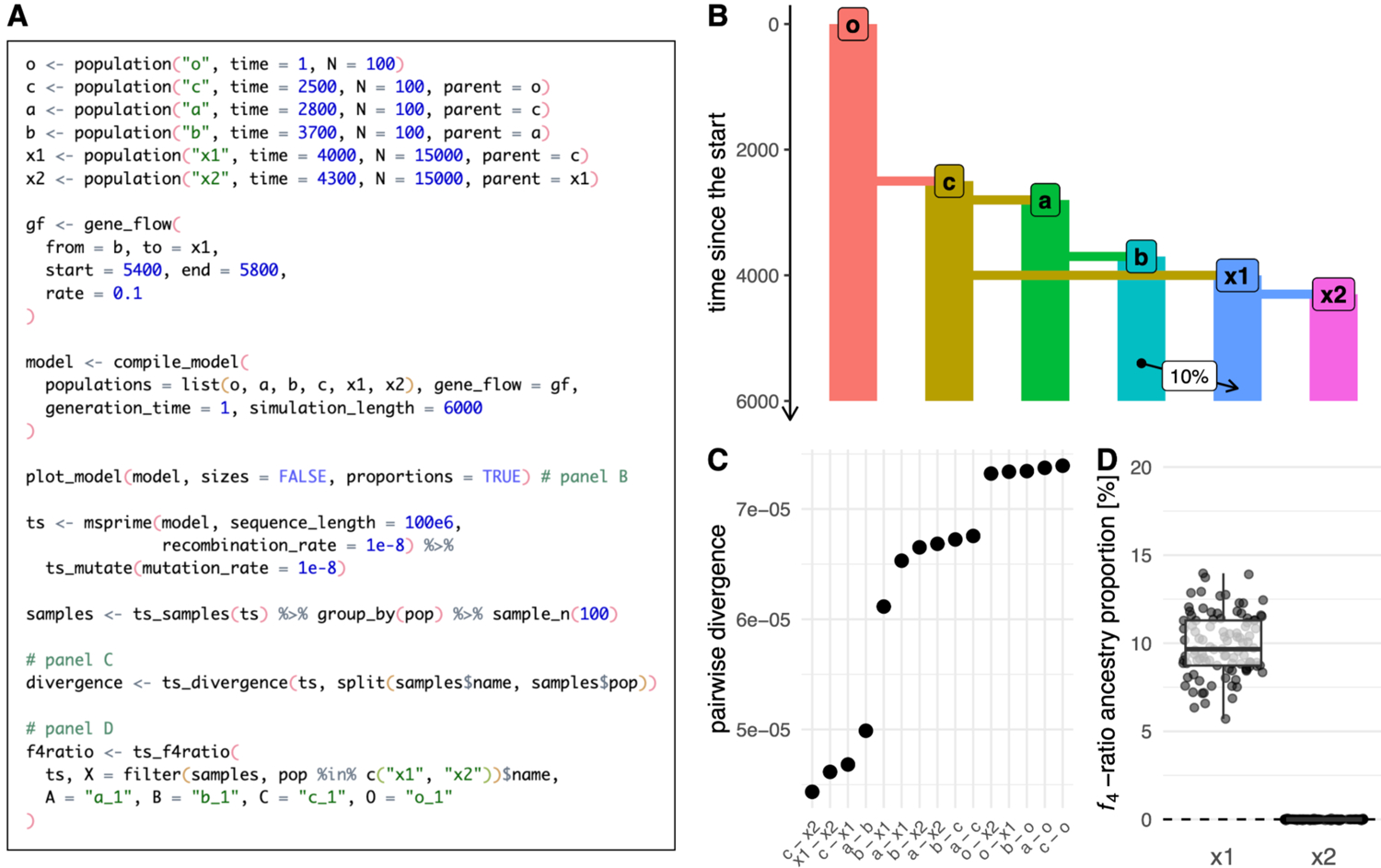
Example 1: specifying a non-spatial model and computing statistics on tree-sequence output. (A) A script which defines a model of a simple demographic history of six populations, simulates it with the *msprime* back end by calling the function msprime(), and performs analyses shown in B–D. (B) A visual overview of the compiled *slendr* model produced by plot_model() prior to simulation. (C) Visualization of the data frame produced by ts_divergence() on the output tree sequence simulated. (D) Ancestry proportions estimated with ts_f4ratio() directly from the tree sequence output. As expected from the model definition, the *f*_4_-ratio statistic estimates indicate ~10% ancestry from “b” in the population “x1”, but 0% ancestry in population “x2”; this agrees with the model overview shown in panel B. Full *ggplot2* visualization code for the figures can be found in a vignette dedicated to this paper at www.slendr.net. The runtime for the simulation and analysis shown in A was ~5 minutes, as measured on a 16” MacBook Pro (2021) equipped with the Apple M1 Pro chip, 32 GB RAM, and running macOS Ventura 13.1.

First, we define an abstract demographic model similar to that which is commonly used in teaching the principles behind the *f*_4_-ratio ancestry proportion estimator (Patterson et al., 2012). In *slendr*, we define the model with a straightforward sequence of population() calls that schedule the order of splits for several populations, taking care of parent–daughter population relationships by providing the appropriate population object as a parent argument when creating each daughter population (Figure 2A). We then schedule a single gene-flow event between the populations *“b”* and *“x1”* by calling the gene_flow() function. After compiling the model with compile_model(), we verify its correctness by visualizing the embedded population relationships with plot_model() (Figure 2B). Although only a single gene_flow() event is featured in this example, more complex gene-flow networks can be specified with *slendr*. Conveniently, strict consistency checks validate each encoded gene-flow event before the computationally costly simulation is run. Examples of complex models with dozens or hundreds of gene-flow events can be found in the documentation available on the *slendr* website (www.slendr.net).

As stated before, *slendr* provides two simulation back ends; here we use the coalescent *msprime* back end to simulate the model, since SLiM’s spatial capabilities are not required for this simple non-spatial model. However, we note that the function slim() could be used in place of the msprime() call to perform the equivalent forward-time simulation just as easily. By default, *slendr* automatically loads the simulated tree-sequence object which can be immediately used for analysis. In this example, we compute the pairwise divergence between random samples of 100 individuals from each population with the function ts_divergence() (Figure 2C). Finally, we use the function ts_f4ratio() to compute the values of the *f*_4_-ratio estimate of *“b”* ancestry in populations *“x1”* and *“x2”*, which differ in whether or not they experienced gene flow from *“b”* (Figure 2D). All other tree sequence analysis functions of *slendr* (Figure 1) can be accessed in the same way. We note that because *slendr* assigns symbolic, permanent names to individuals during sampling, the users can refer to them with these names during tree-sequence operations such as simplification and when computing tree-sequence statistics.

### Example 2: Model with population dispersal dynamics

**Figure 3 -:**
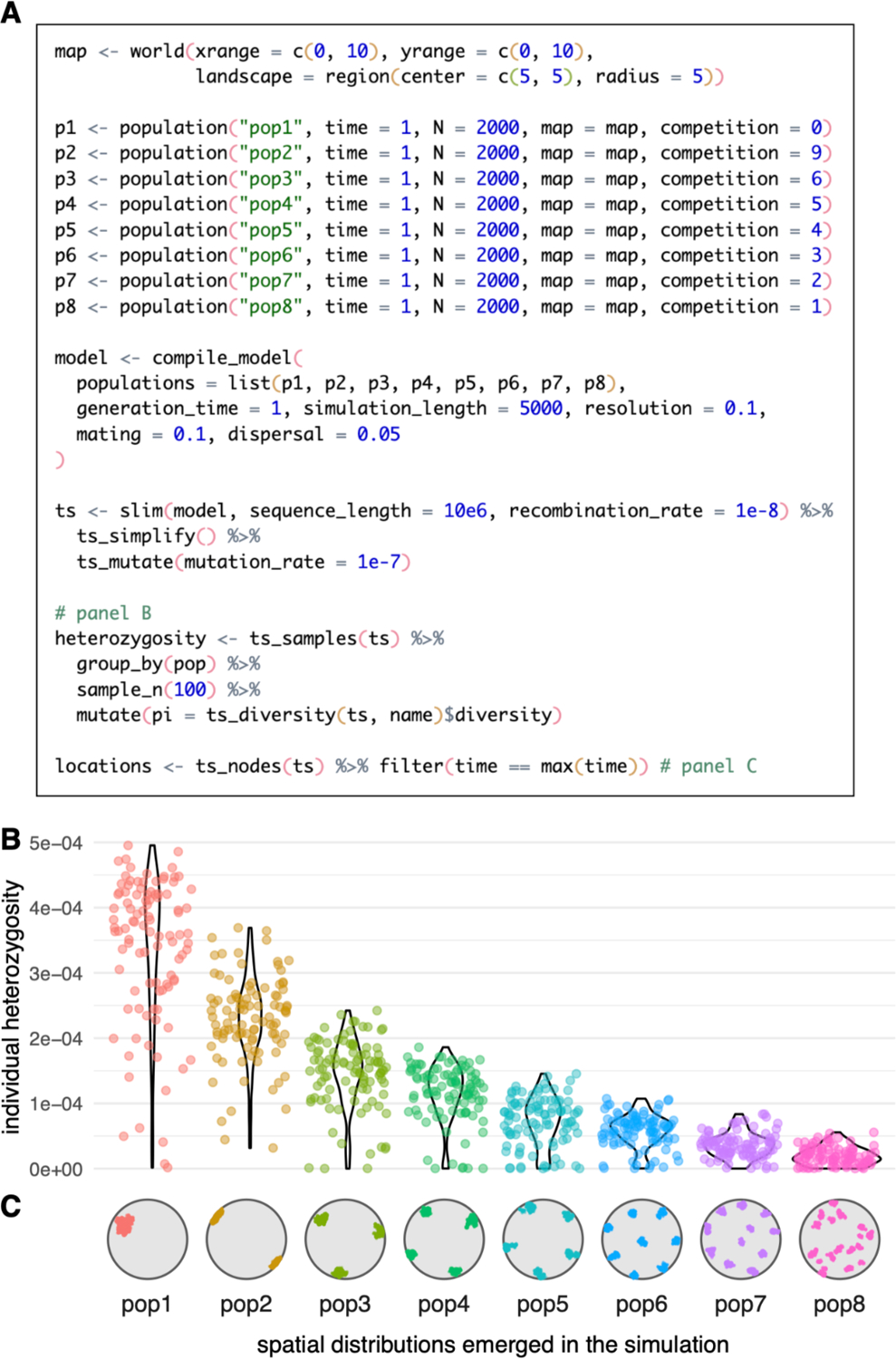
Example 2: a spatial model which involves the parametrization of within-population dispersal dynamics. (A) A complete script which defines eight populations as independent lineages or species, each with constant size and each defined with a different value of *slendr*’s spatial competition parameter, with analysis code to produce panels B–C. The simulation is run with *slendr*’s SLiM back end for 5000 generations, after which a tree sequence recording the genealogical history of 2000×8 diploid individuals is loaded, simplified, and mutated. Heterozygosity is then computed for 100 individuals randomly sampled from each population at the end of simulation. (B) Distribution of heterozygosities of individuals observed in all eight populations. (C) A snapshot of the spatial distributions which emerged as a result of the competition parameter value set for each population. Full visualization code for the figures can be found in a vignette dedicated to this paper at www.slendr.net. The runtime for the simulation and analysis shown in A was ~12 minutes, as measured on a 16” MacBook Pro (2021) equipped with the Apple M1 Pro chip, 32 GB RAM, and running macOS Ventura 13.1.

In our second example, we move from a non-spatial, random-mating model to a model which is explicitly spatial. First, we create an abstract, circular world map using the function world(), producing a completely featureless landscape (see *Example 3* for a more elaborate world map). We then create a series of eight populations which all occupy that map, as specified by the map argument to population(), but do not interact with each other. For simplicity, each population forms its own evolutionary lineage without additional splits or gene-flow events. Importantly, we set the competition parameter of each population to a value which forces the individuals to assume an increasing degree of spatial subdivision which, in turn, affects the amount of diversity expected in each population. Finally, we compile the model to a single object with compile_model() and run it with the slim() back end, simulating 16.000 diploid genomes of 10 megabases each (Figure 3A). After the simulation finishes, we simplify the produced tree sequence, overlay mutations on the simulated genealogies, and use the *slendr* function ts_diversity() to compute the expected heterozygosity in a sample of 100 individuals from each population, inspecting how heterozygosity is affected by the emergent spatial arrangement of each population (Figure 3B, C). We note that some of the values of the spatial competition distance parameter used in this example are quite large, especially compared to the much shorter maximum distance of individual dispersal and mating. Although biologically rather unrealistic, the competition distances have been chosen to give rise to very different degrees of spatial subdivision and, consequently, to varying levels of population genetic diversity, with the intention to demonstrate the ease with which a wide range of model dynamics can be configured by the user.

### Example 3: A toy model of movements and expansions of human populations in West Eurasia over the last 50,000 years

In this example we further expand on the *slendr* functionality demonstrated in the first two examples, introducing programming of expansions and migrations of entire population ranges across a realistic landscape—perhaps the most distinctive feature of *slendr*. The model we implemented here is inspired by large-scale population migrations and turnover events inferred from ancient DNA analyses of human remains from across West Eurasia (Lazaridis et al., 2014; Allentoft et al., 2015; Haak et al., 2015), although we caution that it is an extremely simplified toy model intended only as an illustrative example.

**Figure 4 -:**
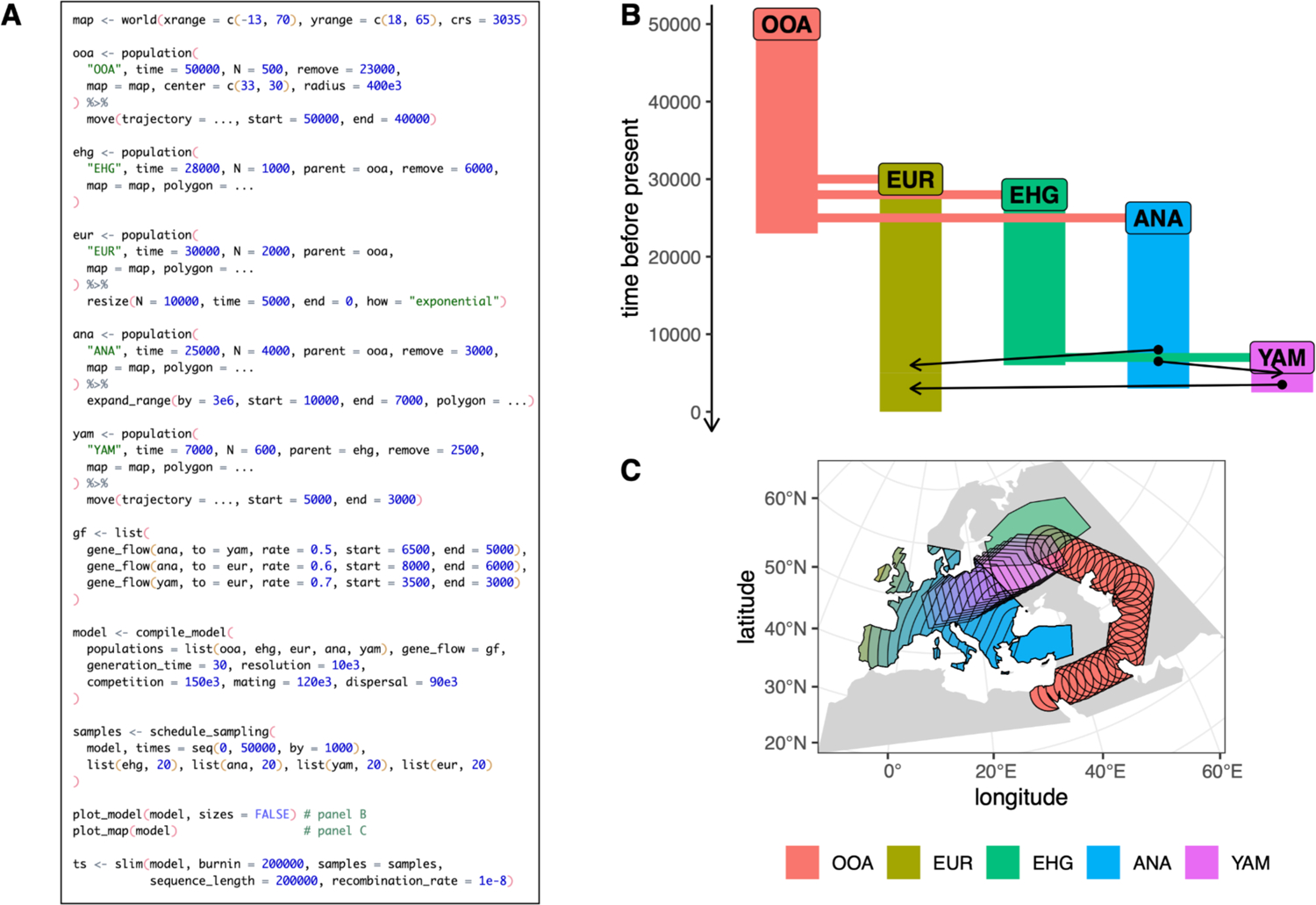
Example 3: a demographic model on a real Earth landscape. (A) A *slendr* script which defines a toy spatio-temporal model of human prehistory in West Eurasia, with analysis code that produces panels B–C. For brevity, we do not specify the full set of coordinates for each spatial demographic event or population range polygon, instead indicating them as ”…”; the complete reproducible code can be found in a vignette dedicated to this paper at www.slendr.net. (B) Visual summary of the non-spatial component of the demographic model, produced by plot_model() with arrows indicating gene flow events. (C) A “compressed” view of spatio-temporal snapshots of population ranges throughout the course of the model prior to the simulation, produced by plot_map(). The runtime for the simulation shown in A was ~3 minutes, as measured on a 16” MacBook Pro (2021) equipped with the Apple M1 Pro chip, 32 GB RAM, and running macOS Ventura 13.1.

Similarly to *Example 2*, we begin by defining a world map for the simulation (Figure 4A), in this case using realistic Earth cartographic data provided by the Natural Earth project (www.naturalearthdata.com). Because we focus on the broad region of West Eurasia, we select the most appropriate coordinate reference system (CRS) for projecting this region on a two-dimensional map which is EPSG:3035. We then define a series of populations, specifying their approximate geographic ranges using simple polygons. We then use the functions move() and expand_range() to schedule when and where populations should migrate, and by what distance and how quickly their population ranges should expand across the landscape during simulation. We again use plot_model() to visualize the demographic history embedded in the *slendr* model as a non-spatial tree-like structure with gene-flow edges (Figure 4B); here, we also use plot_map() to get a “compressed” overview of the spatio-temporal population range dynamics on the simulated map (Figure 4C). We note that unlike in the two previous examples, which were specified in forward time units, this example expresses the timing of demographic events in units of “years before present” which is more natural to this model.

In the previous two code examples (Figure 2A, 3A) we used the default tree-sequence sampling of *slendr*, which implicitly records the genomes of all the diploid individuals alive at the end of a simulation. In this example, we instead use schedule_sampling() to specify a series of sampling events from each population every 1,000 years. We then execute the compiled model and the sampling schedule specified using the slim() back-end, which records only the scheduled set of sampled individuals in the tree-sequence output file.

### Example 4: Visualization of individual trees and spatio-temporal ancestral lineages across a landscape

**Figure 5 -:**
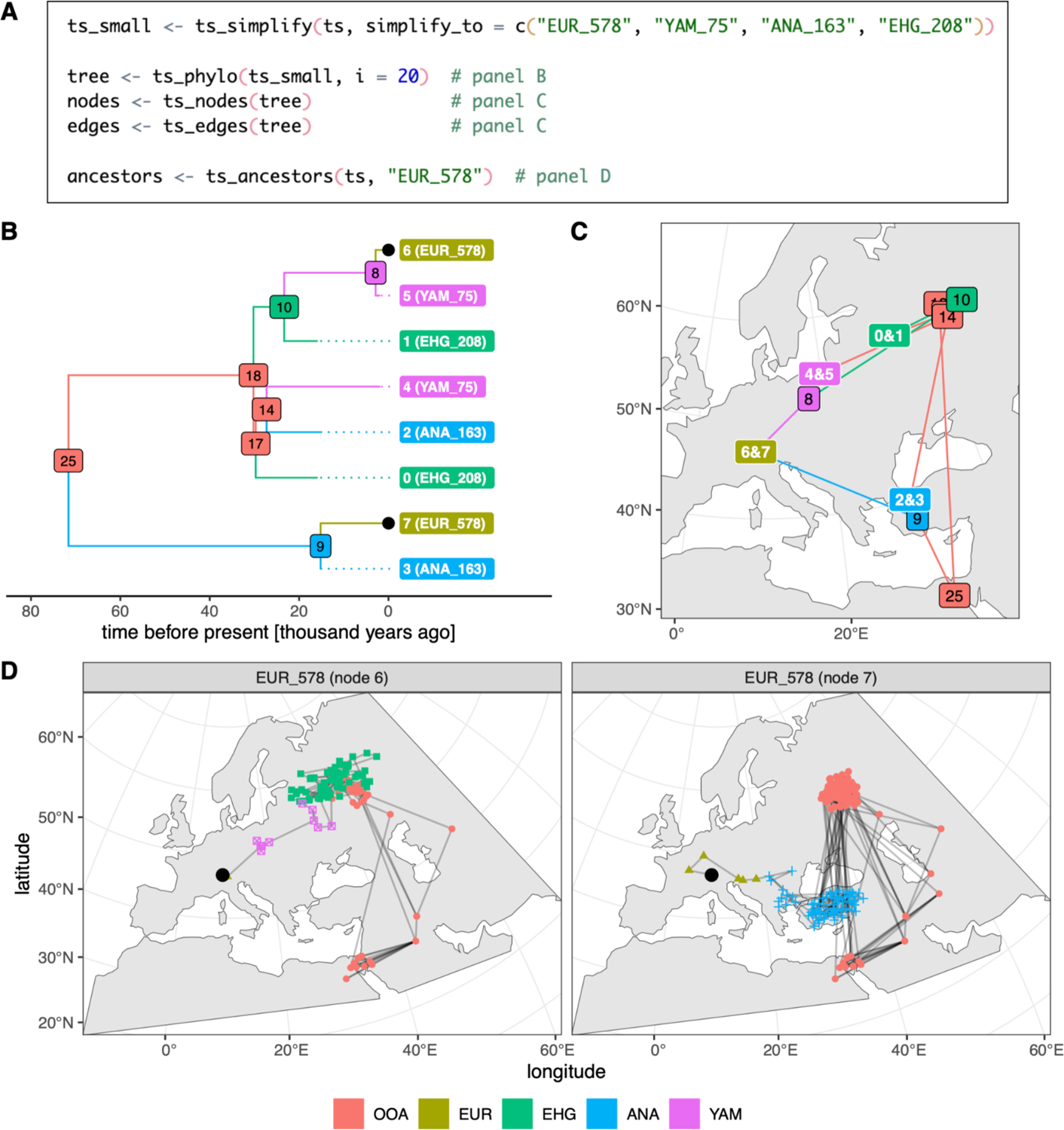
Example 4: accessing and visualizing spatio-temporal information encoded in trees and tree sequences simulated with the *slendr*. (A) A continuation of the script from Example 3, showing how a (potentially very large) tree sequence generated from a *slendr* model can be simplified to a subset of individuals with ts_simplify(). A single tree from the tree sequence is then extracted with ts_phylo(), the tables of spatio-temporal locations of nodes and branches of the tree are extracted by ts_nodes() and ts_edges(), and ancestry information for one individual across the entire tree sequence is extracted with ts_ancestors(), in order to produce the data plotted in B–D. (B) A visualization of the tree extracted by ts_phylo() using standard visualization features of the *ggplot2* and *ggtree* R packages. Dotted lines indicate shortened branches of ancient samples. (C) Visualization of the tree from panel B as a network across the original spatial simulation landscape, with each node indicating the location of a particular individual who lived at some point during the simulation. Labels with two numbers correspond to the locations of sampled individuals, each carrying two chromosomes which are represented by two nodes in the tree sequence. All node numbers correspond to those shown in the tree in panel B. The plot was generated with *ggplot2* using the *sf*-formatted data extracted by ts_nodes() and ts_edges(). (D) A visualization of the spatio-temporal ancestry of a single simulated European individual, “EUR_578”, using the information from the entire tree sequence. Each sub-panel shows the spatial ancestry distribution of one of the two chromosomes carried by this individual (the location of whom is indicated by a black dot), tracing its ancestry through different lineages all the way back to a population in Africa. For easier reference, the same black dots indicate the two chromosomes of this individual also in the tree in panel B. The *ggplot2* code for the figures is omitted for brevity. Full reproducible code examples including the visualization code can be found in a vignette dedicated to this paper at www.slendr.net. The runtime for the code shown in A was ~1 second, as measured on a 16” MacBook Pro (2021) equipped with the Apple M1 Pro chip and 32 GB RAM, running macOS Ventura Version 13.1.

In our final example (Figure 5), we return to the abstract toy model of West Eurasian prehistory developed in Example 3. To leverage *slendr*’s power to simulate genomic data from complex spatial demographic models, *slendr* makes it easy to tap into the large library of geospatial data science packages available for R (Lovelace et al., 2019) by automatically converting simulated spatial locations to an *sf*-compatible tabular format (Pebesma, 2018), as we will see here.

To demonstrate the richness of the spatio-temporal information recorded in the tree sequence, we use the full tree sequence produced by the code in Figure 4A and simplify it so that it contains only the history of a small subset of the thousands of individuals sampled during the spatio-temporal simulation (Figure 5A). We then extract the 20th tree in the tree sequence with *slendr*’s function ts_phylo(), which converts a tree from the *tskit* tree sequence into an R phylo format defined by the *ape* R package, a standard tool for phylogenetics in R (Paradis & Schliep, 2019). Such tree objects can be analyzed by any of the dozens of R packages which operate on *ape*’s phylo trees—for instance, in Figure 5B we show a visualization of this tree using the R package *ggtree* (Yu et al., 2017). Furthermore, because the tree was generated from a spatially-annotated tree sequence, the user can extract information about the location of each individual (or node) in the tree across space and time, as well as ancestral relationships between nodes in the tree, using ts_nodes() and ts_edges() respectively. Crucially, because these functions automatically convert locations into *sf*’s geospatial representation (including the appropriate CRS projection), the results can be immediately plotted on a map with *ggplot2*, which has built-in support for *sf* data (Figure 5C).

In addition to extracting and visualizing single trees representing a genealogy of a set of sampled genomes descending from a common ancestor (spatial or non-spatial), *slendr* also provides a way to extract the complete spatio-temporal ancestry of a single sample going back in time across the entire tree sequence, potentially spanning many trees with thousands of the sample’s ancestors. This can be accomplished with the function ts_ancestors() which, in an analogous way to ts_nodes() and ts_edges(), exposes the spatio-temporal information in the tree sequence as an *sf* object which can be visualized on a map with *ggplot2*. In this example, we use ts_ancestors() to reconstruct the spatio-temporal ancestry distribution for a single simulated European individual (“EUR_578”, represented by the black dot in Figure 5D). Because this individual is diploid, we can trace the ancestry carried by its one chromosome through an expansion from Anatolia (Figure 5D, right panel), while its other chromosome clearly traces its ancestry to a population which migrated to central Europe from an eastern population (Figure 5D, left panel).

Note that by default, the tree sequence output of a *slendr* simulation only contains information about ancestors which are represented by coalescent nodes in some marginal tree—i.e., nodes which are a most recent common ancestor of some pair of sampled nodes. In this example, in Figure 5B and C we can see that the most immediate ancestor (node number 9) of one chromosome of the sampled individual “EUR_578” lived in the region of Anatolia, but the ancestor of its second chromosome lived in Europe (node number 8); but we do not know where all the ancestors along the edges between nodes 9–7 and 8–6, since they were simplified away. Similarly, Figure 5D shows the distributions of locations of most recent common ancestors, not all ancestors. The distribution of ancestors at a particular point in time could be obtained by adding an appropriate sampling event to *slendr*’s sampling schedule and then extracting ancestors from that time.

## Discussion

The *slendr* R package provides a new programmable framework for simulating complex spatio-temporal genomic data. The package implements a set of features for defining spatial population range dynamics with a declarative and visually-focused R interface and uses a tailor-made SLiM script as an efficient population genetic simulation engine. Additionally, *slendr* provides a convenient new way to simulate and analyze large-scale genomic data sets even from traditional, non-spatial demographic models using *msprime* entirely within the R environment.

Owing to its declarative interface, which requires little code even for complex models, the *slendr* package is highly accessible even to researchers or students with little or no prior experience in programming. One of the major challenges for novice population geneticists is having to learn how to integrate multiple different software tools and programming frameworks. R (R Core Team, 2021) is often the first language that biology and bioinformatics students learn, since it offers a large number of libraries for data analysis, statistics, and plotting (Wickham & Grolemund, 2016). For these users, *slendr* provides the opportunity to explore population genetic concepts and simulate realistic population genomic data as soon as they learn the most basic principles of R (i.e., how to call R functions and work with data frames), without first having to learn Python for *msprime* simulations (Baumdicker et al., 2022), shell scripting for simulators from the *ms* family (Hudson, 2002; Staab et al., 2015), or Eidos for SLiM (Haller & Messer, 2019).

Tree sequences provide an efficient way to compute many commonly used population genetic statistics directly on the simulated genealogies (Ralph et al., 2020); because *slendr* uses the tree sequence as its default output format (Kelleher et al., 2018; Haller et al., 2019), in many cases users do not need to convert simulation outputs to external file formats such as VCF or EIGENSTRAT for analysis in other software. This way, *slendr* simulations can be readily used in model fitting and population genetic analyses in situations which have traditionally required converting simulated data to genotype files before analyzing them with population genetics tools such as PLINK (Purcell et al., 2007) or ADMIXTOOLS (Patterson et al., 2012). That said, export to VCF and EIGENSTRAT genotype file formats is supported with a single function call (ts_vcf() and ts_eigenstrat()) if needed.

A key principle in the design of *slendr* has been reproducibility (Sandve et al., 2013): a complete *slendr* simulation and analysis workflow can be written as a single R script. Additionally, the compilation of any *slendr* model produces a self-contained “bundle directory” containing all model configuration files and simulation back-end scripts required to execute the model from the command-line. Although accessing this directory is not necessary for standard workflows because *slendr* operates entirely from R, these bundles can be checked into a git history and provided as supplementary files along with a publication, allowing independent replication even without relying on *slendr* itself.

Moving forward, we expect that the *slendr* framework will become a useful tool to produce ground-truth data for comparing and benchmarking inference methods for modeling spatial genomic processes (Peter & Slatkin, 2013; Petkova et al., 2016; Marcus et al., 2021; Muktupavela et al., 2022), as well as for the development of new approaches to spatial problems in population genomics. There is great potential for deploying *slendr* in simulation-based inference methods, like Approximate Bayesian Computation (ABC) (Beaumont et al., 2002; Csilléry et al., 2010), thanks to its tight integration with the rest of the R modeling landscape. A major challenge in ABC is the significant amount of coding needed to program simulations of demographic history and integrate them with software for computing population genetic statistics. *slendr* can program complex models and compute relevant statistics using its tree-sequence interface with a relatively small amount of code, all within a single R workflow. Furthermore, although *slendr* does not currently include features for implicit, automated parallelism (an important aspect of computation-heavy modeling approaches such as ABC), users can rely on numerous R packages providing a wide range of parallelization techniques (Eddelbuettel, 2021).

Nonetheless, inference of spatial dynamics from genetic data remains an open research problem with many potential pitfalls, and we strongly caution users to avoid overinterpretation. For instance, *slendr* models retain a notion of discretely delineated populations, but even a reasonable fit of such a model to real data does not erase the reality that such groupings are rarely, if ever, as stable and cleanly distinguished as in idealized models. Indeed, confounding the simple models used in population genetics with reality can be actively harmful (Coop, 2022; Khan et al., 2022). Furthermore, population genetic modeling in general is notoriously challenging due to the many parameters involved (Gravel et al., 2011; Pickrell & Pritchard, 2012; Kamm et al., 2020). In this respect, advanced, explicitly spatial models of the kind unlocked by *slendr* present an even bigger challenge. For instance, how can we best do model comparison, and among what set of models? What would constitute a good “null hypothesis” when modeling potentially complex spatial population dynamics? Furthermore, even relatively simple models can be ill-posed or even nonidentifiable: many combinations of spatial parameters (such as individual dispersal or mating distances) may give rise to similar genetic patterns. As every demographic inference study makes assumptions about the process which generated the data, sometimes explicitly and sometimes implicitly, awareness of these assumptions and careful approach to modeling (Beaumont, 2010; Gerbault et al., 2014; Loog, 2021) are vital for correct interpretation of results. We hope that the ease with which *slendr* allows one to explore the impact of spatio-temporal parameters on population dynamics—and the fact that *slendr* forces the researcher to state those parameters explicitly—will help guide researchers in establishing guidelines for good practice, to delineate the limits of what can be learned and, consequently, avoid overinterpretation (or misinterpretation) of such parameters.

In its current version (v0.5.0 as available on the CRAN repository), *slendr*’s spatial simulation maps are limited to landscapes that exhibit binary habitability—i.e., any given location either is or is not habitable by individuals. A more ecologically realistic simulation could allow for varying degrees of habitability at different locations, which would affect the size of the simulated population. Future extensions of the *slendr* framework could include the incorporation of fine-scaled geographic maps storing individual habitability values for each pixel of the raster, allowing for dynamic changes of such maps over time. This would effectively make the size of the population an emergent consequence of the habitability metric aggregated across the map. This extension would require significant changes to the *slendr* back-end code, moving to modeling population densities per unit of landscape area using non-Wright–Fisher dynamics, but the necessary software building blocks are already supported by SLiM and examples of these types of simulations are discussed in the SLiM manual (Haller & Messer, 2023). A recently published Python module Geonomics provides an interface for simulating genetic data on arbitrary landscape rasters (Terasaki Hart et al., 2021). Implementing such functionality in *slendr* would have the advantage of using a much more efficient SLiM simulation engine and a greater ease of use due to *slendr*’s emphasis on visually-focused interactive model design in R. The main challenge would therefore lie in making sure that the additional complexity involved in making the *slendr*’s SLiM back end more flexible does not compromise the current simplicity of its declarative interface. The benefits of this extension would be numerous, including for genomic forecasting and predicting species ranges in the face of climate change and ecological breakdown (Fitzpatrick & Keller, 2015; Exposito-Alonso et al., 2019; Theodoridis et al., 2020), and for constructing models of species distribution dynamics in the ancient past (Wang et al., 2021). Implementation of this extension of *slendr* is still in the planning stages, in collaboration with the community on the project’s GitHub page.

At the moment, *slendr* can only produce genome sequences from a single species (although with an arbitrary number and spatial arrangement of populations of the species) due to the restrictions imposed by its simulation back end. However, many types of genomic resources distributed across space and time are represented by fragmentary mixtures of genomes from multiple species. This includes ancient microbiomes from human remains (Rasmussen et al., 2015), sedimentary DNA from permafrost, caves, or lake and marine cores (Willerslev et al., 2003; Parducci et al., 2017; Armbrecht et al., 2019; Vernot et al., 2021), and environmental DNA from water, soil, or air samples (Taberlet et al., 2012; Stat et al., 2017; Lynggaard et al., 2022). Recent developments in SLiM would allow *slendr* to perform multi-species simulations, which would facilitate ecological modeling of species distributions (Fordham et al., 2021) or of past epidemics (Duchene et al., 2020) from a fully genomic perspective.

Finally, at the time of writing, *slendr* models are limited to neutral simulations, and this restriction applies even to simulations performed via its SLiM back end. In particular, *slendr* does not currently provide built-in support for specifying mutation types, genomic element types, recombination maps, or custom SLiM callbacks. Providing an R equivalent for SLiM’s complete functionality would be a daunting task of limited utility, and would substantially complicate *slendr*’s intuitive R syntax for encoding demographic models (Figure 1). An attractive alternative for supporting more advanced, customized models could be to retain the behavior of slendr described in this manuscript as the default, but provide the possibility of overriding different aspects of this behavior by injecting user-defined SLiM snippets at appropriate locations in *slendr*’s SLiM back-end code. We are exploring this possibility for future versions of the software.

Ultimately, we hope that our new simulation framework will help generate new ideas about the insights that can be gleaned from the rich spatio-temporal information hidden within DNA sequences. Furthermore, we aspire to help budding researchers in population genetics get started with simulations and build their intuition about population genetic concepts by developing models using more traditional non-spatial methods and statistics, and we believe that slendr could be a useful tool for teaching population genetics to students. We hope that by easily generating and visualizing genomic models on real landscapes, we can spark new ways of thinking about how organisms evolve (Bradburd & Ralph, 2019) and enable clearer discussions about the fundamental interconnectedness of genomes across space and time (Mathieson & Scally, 2020).

## References

[R1] Al-Asadi H, Petkova D, Stephens M, Novembre J (2019) Estimating recent migration and population-size surfaces. PLoS genetics, 15, e1007908. 10.1371/journal.pgen.1007908 30640906 PMC6347299

[R2] Allentoft ME, Sikora M, Sjögren K-G, Rasmussen S, Rasmussen M, Stenderup J, Damgaard PB, Schroeder H, Ahlström T, Vinner L (2015) Population genomics of Bronze Age Eurasia. Nature, 522, 167–172. 10.1038/nature14507 26062507

[R3] Armbrecht LH, Coolen MJL, Lejzerowicz F, George SC, Weyrich LS (2019) Ancient DNA from marine sediments: Precautions and considerations for seafloor coring, sample handling and data generation. Earth-Science Reviews, 196, 102887. 10.1016/j.earscirev.2019.102887

[R4] Barton N (1979) Gene flow past a cline. Heredity, 43, 333–339. 10.1038/hdy.1979.86

[R5] Barton N, Depaulis F, Etheridge AM (2002) Neutral evolution in spatially continuous populations. Theoretical population biology, 61, 31–48. 10.1006/tpbi.2001.1557 11895381

[R6] Barton N, Etheridge A, Véber A (2010) A New Model for Evolution in a Spatial Continuum. Electronic Journal of Probability, 15. 10.1214/EJP.v15-741

[R7] Barton N, Etheridge AM, Véber A (2013) Modelling evolution in a spatial continuum. Journal of Statistical Mechanics: Theory and Experiment, 2013, P01002. 10.1088/1742-5468/2013/01/P01002

[R8] Baumdicker F, Bisschop G, Goldstein D, Gower G, Ragsdale AP, Tsambos G, Zhu S, Eldon B, Ellerman EC, Galloway JG (2022) Efficient ancestry and mutation simulation with msprime 1.0. Genetics, 220. 10.1093/genetics/iyab229 PMC9176297 34897427

[R9] Beaumont MA (2010) Approximate Bayesian Computation in Evolution and Ecology. Annual review of ecology, evolution, and systematics, 41, 379–406. 10.1146/annurev-ecolsys-102209-144621

[R10] Beaumont MA, Zhang W, Balding DJ (2002) Approximate Bayesian computation in population genetics. Genetics, 162, 2025–2035. 10.1093/genetics/162.4.2025 12524368 PMC1462356

[R11] Beerli P, Felsenstein J (2001) Maximum likelihood estimation of a migration matrix and effective population sizes in n subpopulations by using a coalescent approach. Proceedings of the National Academy of Sciences of the United States of America, 98, 4563–4568. 10.1073/pnas.081068098 11287657 PMC31874

[R12] Bergström A, Frantz L, Schmidt R, Ersmark E, Lebrasseur O, Girdland-Flink L, Lin AT, Storå J, Sjögren KG, Anthony D (2020) Origins and genetic legacy of prehistoric dogs. Science, 370, 557–564. 10.1126/science.aba9572 33122379 PMC7116352

[R13] Bradburd GS, Coop GM, Ralph PL (2018) Inferring Continuous and Discrete Population Genetic Structure Across Space. Genetics, 210, 33–52. 10.1534/genetics.118.301333 30026187 PMC6116973

[R14] Bradburd GS, Ralph PL (2019) Spatial population genetics: It’s about time. Annual review of ecology, evolution, and systematics, 50, 427–449. 10.1146/annurev-ecolsys-110316-022659

[R15] Chambers JM (2020) S, R, and data science. Proceedings of the ACM on Programming Languages, 4, 1–17. 10.1145/3386334

[R16] Chang W, Cheng J, Allaire J, Sievert C, Schloerke B, Xie Y, Allen J, McPherson J, Dipert A, Borges B (2023). shiny: Web Application Framework for R. R package version 1.8.0.9000, https://github.com/rstudio/shiny, https://shiny.posit.co/.

[R17] Coop G (2022) Genetic similarity versus genetic ancestry groups as sample descriptors in human genetics. arXiv. 10.48550/arXiv.2207.11595

[R18] Crabtree SA, White DA, Bradshaw CJA, Saltré F, Williams AN, Beaman RJ, Bird MI, Ulm S (2021) Landscape rules predict optimal superhighways for the first peopling of Sahul. Nature human behaviour, 5, 1303–1313. 10.1038/s41562-021-01106-8 33927367

[R19] Csilléry K, Blum MGB, Gaggiotti OE, François O (2010) Approximate Bayesian Computation (ABC) in practice. Trends in ecology & evolution, 25, 410–418. 10.1016/j.tree.2010.04.001 20488578

[R20] Currat M, Arenas M, Quilodràn CS, Excoffier L, Ray N (2019) SPLATCHE3: simulation of serial genetic data under spatially explicit evolutionary scenarios including long-distance dispersal. Bioinformatics, 35, 4480–4483. 10.1093/bioinformatics/btz311 31077292 PMC6821363

[R21] Currat M, Excoffier L (2011) Strong reproductive isolation between humans and Neanderthals inferred from observed patterns of introgression. Proceedings of the National Academy of Sciences, 108, 15129–15134. 10.1073/pnas.1107450108 PMC3174651 21911389

[R22] Currat M, Ray N, Excoffier L (2004) splatche: a program to simulate genetic diversity taking into account environmental heterogeneity. Molecular ecology notes, 4, 139–142. 10.1046/j.1471-8286.2003.00582.x

[R23] Danecek P, Auton A, Abecasis G, Albers CA, Banks E, DePristo MA, Handsaker RE, Lunter G, Marth GT, Sherry ST (2011) The variant call format and VCFtools. Bioinformatics, 27, 2156–2158. 10.1093/bioinformatics/btr330 21653522 PMC3137218

[R24] Delser PM, Krapp M, Beyer R, Jones ER, Miller EF, Hovhannisyan A, Parker M, Siska V, Vizzari MT, Pearmain EJ (2021) Climate and mountains shaped human ancestral genetic lineages. bioRxiv, 2021.07.13.452067. 10.1101/2021.07.13.452067

[R25] Duchene S, Featherstone L, Haritopoulou-Sinanidou M, Rambaut A, Lemey P, Baele G (2020) Temporal signal and the phylodynamic threshold of SARS-CoV-2. Virus evolution, 6, veaa061. 10.1093/ve/veaa061 33235813 PMC7454936

[R26] Duforet-Frebourg N, Blum MGB (2014) Nonstationary patterns of isolation-by-distance: inferring measures of local genetic differentiation with Bayesian kriging. Evolution, 68, 1110–1123. 10.1111/evo.12342 24372175 PMC4285919

[R27] Eddelbuettel D (2021) Parallel computing with R: A brief review. Wiley interdisciplinary reviews. Computational statistics, 13. 10.1002/wics.1515

[R28] Ewing G, Hermisson J (2010) MSMS: a coalescent simulation program including recombination, demographic structure and selection at a single locus. Bioinformatics, 26, 2064–2065. 10.1093/bioinformatics/btq322 20591904 PMC2916717

[R29] Exposito-Alonso M, 500 Genomes Field Experiment Team, Burbano HA, Bossdorf O, Nielsen R, Weigel D (2019) Natural selection on the Arabidopsis thaliana genome in present and future climates. Nature, 573, 126–129. 10.1038/s41586-019-1520-9 31462776

[R30] Felsenstein J (1975) A Pain in the Torus: Some Difficulties with Models of Isolation by Distance. The American naturalist, 109, 359–368. 10.1086/283003

[R31] Feuerborn TR, Carmagnini A, Losey RJ, Nomokonova T, Askeyev A, Askeyev I, Askeyev O, Antipina EE, Appelt M, Bachura OP (2021) Modern Siberian dog ancestry was shaped by several thousand years of Eurasian-wide trade and human dispersal. Proceedings of the National Academy of Sciences of the United States of America, 118. 10.1073/pnas.2100338118 PMC8488619 34544854

[R32] Fisher RA (1937) The wave of advance of advantageous genes. Annals of eugenics, 7, 355–369. 10.1111/j.1469-1809.1937.tb02153.x

[R33] Fitzpatrick MC, Keller SR (2015) Ecological genomics meets community-level modelling of biodiversity: mapping the genomic landscape of current and future environmental adaptation. Ecology letters, 18, 1–16. 10.1111/ele.12376 25270536

[R34] Fordham DA, Haythorne S, Brown SC, Buettel JC, Brook BW (2021) poems: R package for simulating species’ range dynamics using pattern-oriented validation. Methods in ecology and evolution / British Ecological Society, 12, 2364–2371. 10.1111/2041-210x.13720

[R35] Frachetti MD, Smith CE, Traub CM, Williams T (2017) Nomadic ecology shaped the highland geography of Asia’s Silk Roads. Nature, 543, 193–198. 10.1038/nature21696 28277506

[R36] Fu Q, Posth C, Hajdinjak M, Petr M, Mallick S, Fernandes D, Furtwängler A, Haak W, Meyer M, Mittnik A (2016) The genetic history of Ice Age Europe. Nature, 534, 200–205. 10.1038/nature17993 27135931 PMC4943878

[R37] Gerbault P, Allaby RG, Boivin N, Rudzinski A, Grimaldi IM, Pires JC, Climer Vigueira C, Dobney K, Gremillion KJ, Barton L (2014) Storytelling and story testing in domestication. Proceedings of the National Academy of Sciences of the United States of America, 111, 6159–6164. 10.1073/pnas.1400425111 24753572 PMC4035932

[R38] Gravel S, Henn BM, Gutenkunst RN, (2011) Demographic history and rare allele sharing among human populations. Proceedings of the National Academy of Sciences, 108, 11983–11988. 10.1073/pnas.1019276108 PMC3142009 21730125

[R39] Green RE, Krause J, Briggs AW, Maricic T, Stenzel U, Kircher M, Patterson N, Li H, Zhai W, Fritz MH (2010) A draft sequence of the Neandertal genome. Science, 328, 710–722. 10.1126/science.1188021 20448178 PMC5100745

[R40] Guillot G, Leblois R, Coulon A, Frantz AC (2009) Statistical methods in spatial genetics. Molecular ecology, 18, 4734–4756. 10.1111/j.1365-294X.2009.04410.x 19878454

[R41] Haak W, Lazaridis I, Patterson N, Rohland N, Mallick S, Llamas B, Brandt G, Nordenfelt S, Harney E, Stewardson K (2015) Massive migration from the steppe was a source for Indo-European languages in Europe. Nature, 522, 207–211. 10.1038/nature14317 25731166 PMC5048219

[R42] Haller BC, Galloway J, Kelleher J, Messer PW, Ralph PL (2019) Tree-sequence recording in SLiM opens new horizons for forward-time simulation of whole genomes. Molecular ecology resources, 19, 552–566. 10.1111/1755-0998.12968 30565882 PMC6393187

[R43] Haller BC, Messer PW (2017) SLiM 2: Flexible, Interactive Forward Genetic Simulations. Molecular biology and evolution, 34, 230–240. 10.1093/molbev/msw211 27702775

[R44] Haller BC, Messer PW (2019) SLiM 3: Forward Genetic Simulations Beyond the Wright-Fisher Model. Molecular biology and evolution, 36, 632–637. 10.1093/molbev/msy228 30517680 PMC6389312

[R45] Haller BC, Messer PW (2023) SLiM 4: Multispecies Eco-Evolutionary Modeling. The American naturalist, 201, E127–E139. 10.1086/723601 PMC10793872 37130229

[R46] Hanks EM, Hooten MB (2013) Circuit theory and model-based inference for landscape connectivity. Journal of the American Statistical Association, 108, 22–33. 10.1080/01621459.2012.724647

[R47] Hudson RR (2002) Generating samples under a Wright-Fisher neutral model of genetic variation. Bioinformatics, 18, 337–338. 10.1093/bioinformatics/18.2.337 11847089

[R48] Kamm J, Terhorst J, Durbin R, Song YS (2020) Efficiently inferring the demographic history of many populations with allele count data. Journal of the American Statistical Association, 115, 1472–1487. 10.1080/01621459.2019.1635482 33012903 PMC7531012

[R49] Kelleher J, Etheridge AM, Barton NH (2014) Coalescent simulation in continuous space: algorithms for large neighbourhood size. Theoretical population biology, 95, 13–23. 10.1016/j.tpb.2014.05.001 24910324

[R50] Kelleher J, Etheridge AM, McVean G (2016) Efficient Coalescent Simulation and Genealogical Analysis for Large Sample Sizes. PLoS computational biology, 12, e1004842. 10.1371/journal.pcbi.1004842 27145223 PMC4856371

[R51] Kelleher J, Thornton KR, Ashander J, Ralph PL (2018) Efficient pedigree recording for fast population genetics simulation. PLoS computational biology, 14, e1006581. 10.1371/journal.pcbi.1006581 30383757 PMC6233923

[R52] Kelleher J, Wong Y, Wohns AW, Fadil C, Albers PK, McVean G (2019) Inferring whole-genome histories in large population datasets. Nature genetics, 51, 1330–1338. 10.1038/s41588-019-0483-y 31477934 PMC6726478

[R53] Khan AT, Gogarten SM, McHugh CP, Stilp AM, Sofer T, Bowers ML, Wong Q, Cupples LA, Hidalgo B, Johnson AD (2022) Recommendations on the use and reporting of race, ethnicity, and ancestry in genetic research: Experiences from the NHLBI TOPMed program. Cell genomics, 2. 10.1016/j.xgen.2022.100155 PMC9481067 36119389

[R54] Lazaridis I, Patterson N, Mittnik A, Renaud G, Mallick S, Kirsanow K, Sudmant PH, Schraiber JG, Castellano S, Lipson M (2014) Ancient human genomes suggest three ancestral populations for present-day Europeans. Nature, 513, 409–413. 10.1038/nature13673 25230663 PMC4170574

[R55] Levene H (1953) Genetic Equilibrium When More Than One Ecological Niche is Available. The American naturalist, 87, 331–333. 10.1086/281792

[R56] Librado P, Khan N, Fages A, Kusliy MA, Suchan T, Tonasso-Calvière L, Schiavinato S, Alioglu D, Fromentier A, Perdereau A (2021) The origins and spread of domestic horses from the Western Eurasian steppes. Nature, 1–7. 10.1038/s41586-021-04018-9 PMC8550961 34671162

[R57] Liu H, Prugnolle F, Manica A, Balloux F (2006) A geographically explicit genetic model of worldwide human-settlement history. American journal of human genetics, 79, 230–237. 10.1086/505436 16826514 PMC1559480

[R58] Loog L (2021) Sometimes hidden but always there: the assumptions underlying genetic inference of demographic histories. Philosophical transactions of the Royal Society of London. Series B, Biological sciences, 376, 20190719. 10.1098/rstb.2019.0719 33250022 PMC7741104

[R59] Loog L, Mirazón Lahr M, Kovacevic M, Manica A, Eriksson A, Thomas MG (2017) Estimating mobility using sparse data: Application to human genetic variation. Proceedings of the National Academy of Sciences, 114, 12213–12218. 10.1073/pnas.1703642114 PMC5699029 29087301

[R60] Lovelace R, Nowosad J, Muenchow J (2019) Geocomputation with R. Routledge. Chapman and Hall/CRC, New York. 10.1201/9780203730058

[R61] Lynggaard C, Bertelsen MF, Jensen CV, Johnson MS, Frøslev TG, Olsen MT, Bohmann K (2022) Airborne environmental DNA for terrestrial vertebrate community monitoring. Current biology: CB, 32, 701–707.e5. 10.1016/j.cub.2021.12.014 34995490 PMC8837273

[R62] Malécot G (1951) Un traitement stochastique des problèmes linéaires en génétique de population. Ann. Univ. Lyon. Sci. Sec, 14, 79–117.

[R63] Mallick S, Li H, Lipson M, Mathieson I, Gymrek M, Racimo F, Zhao M, Chennagiri N, Nordenfelt S, Tandon A (2016) The Simons Genome Diversity Project: 300 genomes from 142 diverse populations. Nature, 538, 201–206. 10.1038/nature18964 27654912 PMC5161557

[R64] Marcus J, Ha W, Barber RF, Novembre J (2021) Fast and flexible estimation of effective migration surfaces. eLife, 10. 10.7554/eLife.61927 PMC8324296 34328078

[R65] Mathieson I, Scally A (2020) What is ancestry. PLoS genetics, 16, e1008624. 10.1371/journal.pgen.1008624 32150538 PMC7082057

[R66] McRae BH (2006) Isolation by resistance. Evolution, 60, 1551–1561. 10.1111/j.0014-3820.2006.tb00500.x 17017056

[R67] Meyer M, Palkopoulou E, Baleka S, Stiller M, Penkman KEH, Alt KW, Ishida Y, Mania D, Mallick S, Meijer T (2017) Palaeogenomes of Eurasian straight-tusked elephants challenge the current view of elephant evolution. eLife, 6, e25413. 10.7554/eLife.25413 28585920 PMC5461109

[R68] Muktupavela RA, Petr M, Ségurel L, Korneliussen T, Novembre J, Racimo F (2022) Modeling the spatiotemporal spread of beneficial alleles using ancient genomes. eLife, 11, e73767. 10.7554/eLife.73767 36537881 PMC9767474

[R69] Nagylaki T (1976) The relation between distant individuals in geographically structured populations. Mathematical biosciences, 28, 73–80. 10.1016/0025-5564(76)90096-1

[R70] Osmond MM, Coop G (2021) Estimating dispersal rates and locating genetic ancestors with genome-wide genealogies. bioRxiv, 2021.07.13.452277. 10.1101/2021.07.13.452277 PMC11658769 39589398

[R71] Palkopoulou E, Lipson M, Mallick S, Nielsen S, Rohland N, Baleka S, Karpinski E, Ivancevic AM, To TH, Kortschak RD (2018) A comprehensive genomic history of extinct and living elephants. Proceedings of the National Academy of Sciences of the United States of America, 115, E2566–E2574. 10.1073/pnas.1720554115 29483247 PMC5856550

[R72] Paradis E (2012) Analysis of Phylogenetics and Evolution with R. Springer, New York, NY. 10.1007/978-1-4614-1743-9

[R73] Paradis E, Schliep K (2019) ape 5.0: an environment for modern phylogenetics and evolutionary analyses in R (Schwartz R, Ed,). Bioinformatics, 35, 526–528. 10.1093/bioinformatics/bty633 30016406

[R74] Parducci L, Bennett KD, Ficetola GF, Alsos IG, Suyama Y, Wood JR, Pedersen MW (2017) Ancient plant DNA in lake sediments. The New phytologist, 214, 924–942. 10.1111/nph.14470 28370025

[R75] Patterson N, Moorjani P, Luo Y, Mallick S, Rohland N, Zhan Y, Genschoreck T, Webster T, Reich D (2012) Ancient admixture in human history. Genetics, 192, 1065–1093. 10.1534/genetics.112.145037 22960212 PMC3522152

[R76] Pebesma E (2018) Simple features for R: Standardized support for spatial vector data. The R journal, 10, 439. 10.32614/rj-2018-009

[R77] Peter BM, Slatkin M (2013) Detecting range expansions from genetic data. Evolution, 67, 3274–3289. 10.1111/evo.12202 24152007 PMC4282923

[R78] Petkova D, Novembre J, Stephens M (2016) Visualizing spatial population structure with estimated effective migration surfaces. Nature genetics, 48, 94–100. 10.1038/ng.3464 26642242 PMC4696895

[R79] Petr M (2023). slendr GitHub repository (Peer Community Journal archive). Zenodo. 10.5281/zenodo.10384289

[R80] Pickrell JK, Pritchard JK (2012) Inference of Population Splits and Mixtures from Genome-Wide Allele Frequency Data (Tang H, Ed,). PLoS genetics, 8, e1002967. 10.1371/journal.pgen.1002967 23166502 PMC3499260

[R81] Pickrell JK, Reich D (2014) Toward a new history and geography of human genes informed by ancient DNA. Trends in genetics: TIG, 30, 377–389. 10.1016/j.tig.2014.07.007 25168683 PMC4163019

[R82] Purcell S, Neale B, Todd-Brown K, Thomas L, Ferreira MAR, Bender D, Maller J, Sklar P, de Bakker PIW, Daly MJ, Sham PC (2007) PLINK: a tool set for whole-genome association and population-based linkage analyses. American journal of human genetics, 81, 559–575. 10.1086/519795 17701901 PMC1950838

[R83] Racimo F, Woodbridge J, Fyfe RM, Sikora M, Sjögren KG, Kristiansen K, Vander Linden M (2020) The spatiotemporal spread of human migrations during the European Holocene. Proceedings of the National Academy of Sciences of the United States of America, 117, 8989–9000. 10.1073/pnas.1920051117 32238559 PMC7183159

[R84] Ralph P, Coop G (2013) The Geography of Recent Genetic Ancestry across Europe (Tyler-Smith C, Ed,). PLoS biology, 11, e1001555. 10.1371/journal.pbio.1001555 23667324 PMC3646727

[R85] Ralph P, Thornton K, Kelleher J (2020) Efficiently Summarizing Relationships in Large Samples: A General Duality Between Statistics of Genealogies and Genomes. Genetics, 215, 779–797. 10.1534/genetics.120.303253 32357960 PMC7337078

[R86] Rasmussen S, Allentoft ME, Nielsen K, Orlando L, Sikora M, Sjögren K-G, Pedersen AG, Schubert M, Van Dam A, Kapel CMO (2015) Early divergent strains of Yersinia pestis in Eurasia 5,000 years ago. Cell, 163, 571–582. 10.1016/j.cell.2015.10.009 26496604 PMC4644222

[R87] Rasmussen M, Li Y, Lindgreen S, Pedersen JS, Albrechtsen A, Moltke I, Metspalu M, Metspalu E, Kivisild T, Gupta R (2010) Ancient human genome sequence of an extinct Palaeo-Eskimo. Nature, 463, 757–762. 10.1038/nature08835 20148029 PMC3951495

[R88] R Core Team (2021) R: A Language and Environment for Statistical Computing. R Foundation for Statistical Computing, Vienna. https://www.R-project.org

[R89] Ringbauer H, Kolesnikov A, Field DL, Barton NH (2018) Estimating Barriers to Gene Flow from Distorted Isolation-by-Distance Patterns. Genetics, 208, 1231–1245. 10.1534/genetics.117.300638 29311149 PMC5844333

[R90] Rousset F (1997) Genetic differentiation and estimation of gene flow from F-statistics under isolation by distance. Genetics, 145, 1219–1228. 10.1093/genetics/145.4.1219 9093870 PMC1207888

[R91] Safner T, Miller MP, McRae BH, Fortin M-J, Manel S (2011) Comparison of Bayesian clustering and edge detection methods for inferring boundaries in landscape genetics. International journal of molecular sciences, 12, 865–889. 10.3390/ijms12020865 21541031 PMC3083678

[R92] Sandve GK, Nekrutenko A, Taylor J, Hovig E (2013) Ten simple rules for reproducible computational research. PLoS computational biology, 9, e1003285. 10.1371/journal.pcbi.1003285 24204232 PMC3812051

[R93] Slatkin M (1973) Gene flow and selection in a cline. Genetics, 75, 733–756. 10.1093/genetics/75.4.733 4778791 PMC1213045

[R94] Slatkin M, Excoffier L (2012) Serial founder effects during range expansion: a spatial analog of genetic drift. Genetics, 191, 171–181. 10.1534/genetics.112.139022 22367031 PMC3338258

[R95] Slatkin M, Racimo F (2016) Ancient DNA and human history. Proceedings of the National Academy of Sciences, 113, 6380–6387. 10.1073/pnas.1524306113 PMC4988579 27274045

[R96] Speidel L, Forest M, Shi S, Myers SR (2019) A method for genome-wide genealogy estimation for thousands of samples. Nature genetics, 51, 1321–1329. 10.1038/s41588-019-0484-x 31477933 PMC7610517

[R97] Staab PR, Zhu S, Metzler D, Lunter G (2015) scrm: efficiently simulating long sequences using the approximated coalescent with recombination. Bioinformatics, 31, 1680–1682. 10.1093/bioinformatics/btu861 25596205 PMC4426833

[R98] Stat M, Huggett MJ, Bernasconi R, DiBattista JD, Berry TE, Newman SJ, Harvey ES, Bunce M (2017) Ecosystem biomonitoring with eDNA: metabarcoding across the tree of life in a tropical marine environment. Scientific reports, 7, 12240. 10.1038/s41598-017-12501-5 28947818 PMC5612959

[R99] Taberlet P, Coissac E, Pompanon F, Brochmann C, Willerslev E (2012) Towards next-generation biodiversity assessment using DNA metabarcoding. Molecular ecology, 21, 2045–2050. 10.1111/j.1365-294X.2012.05470.x 22486824

[R100] Terasaki Hart DE, Bishop AP, Wang IJ (2021) Geonomics: Forward-Time, Spatially Explicit, and Arbitrarily Complex Landscape Genomic Simulations. Molecular biology and evolution, 38, 4634–4646. 10.1093/molbev/msab175 34117771 PMC8476160

[R101] The 1000 Genomes Project Consortium (2010) A map of human genome variation from population-scale sequencing. Nature, 467, 1061–1073. 10.1038/nature09534 20981092 PMC3042601

[R102] Theodoridis S, Fordham DA, Brown SC, Li S, Rahbek C, Nogues-Bravo D (2020) Evolutionary history and past climate change shape the distribution of genetic diversity in terrestrial mammals. Nature communications, 11, 2557. 10.1038/s41467-020-16449-5 PMC7244709 32444801

[R103] Trucchi E (2023) A new powerful tool to easily encode the geo-spatial dimension in population genetics simulations. Peer Community in Evolutionary Biology, 1, 100630. 10.24072/pci.evolbiol.100630

[R104] Vernot B, Zavala EI, Gómez-Olivencia A, Jacobs Z, Slon V, Mafessoni F, Romagné F, Pearson A, Petr M, Sala N (2021) Unearthing Neanderthal population history using nuclear and mitochondrial DNA from cave sediments. Science, 372. 10.1126/science.abf1667 33858989

[R105] Wang Y, Pedersen MW, Alsos IG, De Sanctis B, Racimo F, Prohaska A, Coissac E, Owens HL, Merkel MKF, Fernandez-Guerra A (2021) Late Quaternary dynamics of Arctic biota from ancient environmental genomics. Nature, 600, 86–92. 10.1038/s41586-021-04016-x 34671161 PMC8636272

[R106] Wickham H, Averick M, Bryan J, Chang W, McGowan LD, François R, Grolemund G, Hayes A, Henry L, Hester J (2019) Welcome to the tidyverse. Journal of Open Source Software, 4, 1686. 10.21105/joss.01686

[R107] Wickham H, Grolemund G (2016) R for Data Science. O’Reilly Media, Inc. https://r4ds.had.co.nz/

[R108] Willerslev E, Hansen AJ, Binladen J, Brand TB, Gilbert MTP, Shapiro B, Bunce M, Wiuf C, Gilichinsky DA, Cooper A (2003) Diverse plant and animal genetic records from Holocene and Pleistocene sediments. Science, 300, 791–795. 10.1126/science.1084114 12702808

[R109] Wohns AW, Wong Y, Jeffery B, Akbari A, Mallick S, Pinhasi R, Patterson N, Reich D, Kelleher J, McVean G (2022) A unified genealogy of modern and ancient genomes. Science, 375, eabi8264. 10.1126/science.abi8264 35201891 PMC10027547

[R110] Wright S (1943) Isolation by Distance. Genetics, 28, 114–138. 10.1093/genetics/28.2.114 17247074 PMC1209196

[R111] Yu G, Smith DK, Zhu H, Guan Y, Lam TT-Y (2017) ggtree: An R package for visualization and annotation of phylogenetic trees with their covariates and other associated data. Methods in ecology and evolution / British Ecological Society, 8, 28–36. 10.1111/2041-210x.12628

